# Neural correlates of positive and negative symptoms through the illness course: an fMRI study in early psychosis and chronic schizophrenia

**DOI:** 10.1038/s41598-019-51023-0

**Published:** 2019-10-08

**Authors:** Lucy D. Vanes, Elias Mouchlianitis, Krisna Patel, Erica Barry, Katie Wong, Megan Thomas, Timea Szentgyorgyi, Dan Joyce, Sukhwinder Shergill

**Affiliations:** 1 Wellcome Centre for Human Neuroimaging, University College London, 12 Queen Square, London, WC1N 3AR United Kingdom; 2 Institute of Psychiatry, Psychology and Neuroscience, de Crespigny Park, London, SE5 8AF United Kingdom; 3 Institute Department of Clinical Sciences, Cornell University College of Veterinary Medicine, Ithaca, NY USA

**Keywords:** Cognitive control, Psychosis

## Abstract

Psychotic illness is associated with cognitive control deficits and abnormal recruitment of neural circuits subserving cognitive control. It is unclear to what extent this dysfunction underlies the development and/or maintenance of positive and negative symptoms typically observed in schizophrenia. In this study we compared fMRI activation on a standard Stroop task and its relationship with positive and negative symptoms in early psychosis (EP, N = 88) and chronic schizophrenia (CHR-SZ, N = 38) patients. CHR-SZ patients showed reduced frontal, striatal, and parietal activation across incongruent and congruent trials compared to EP patients. Higher positive symptom severity was associated with reduced activation across both trial types in supplementary motor area (SMA), middle temporal gyrus and cerebellum in EP, but not CHR-SZ patients. Higher negative symptom severity was associated with reduced cerebellar activation in EP, but not in CHR-SZ patients. A negative correlation between negative symptoms and activation in SMA and precentral gyrus was observed in EP patients and in CHR-SZ patients. The results suggest that the neural substrate of positive symptoms changes with illness chronicity, and that cognitive control related neural circuits may be most relevant in the initial development phase of positive symptoms. These findings also highlight a changing role for the cerebellum in the development and later maintenance of both positive and negative symptoms.

## Introduction

Deficits in executive functions are a core characteristic of psychotic disorders. Patients with psychosis typically display impaired performance on tasks involving executive functions such as cognitive control, working memory, planning, and cognitive flexibility^1–3^. Abnormal cognitive control in particular has been proposed as a unifying theory of cognitive deficits in schizophrenia^4^ given its importance in a wider array of higher cognitive domains implicated in the illness, including episodic and working memory, flexible learning, task switching, response inhibition, and emotion regulation^5–10^. Moreover, symptoms of psychosis can be understood in terms of concepts relevant to cognitive control, namely deficits in the flexible, adaptive control of thoughts and behaviour, as well as a difficulty in separating relevant from irrelevant environmental stimuli. Empirically, impaired behaviour on cognitive control tasks has been shown to correlate with symptoms of psychosis^11–15^.

Functional imaging studies have demonstrated that cognitive control deficits in psychosis are accompanied by altered neural activation in relevant brain regions, including anterior cingulate cortex (ACC), supplementary motor area (SMA) and pre-SMA, superior and lateral prefrontal cortex (PFC), parietal cortex and aspects of the basal ganglia^1,4,15–18^. It has been suggested that abnormal recruitment of these neural circuits, particularly in the context of cognitive control processes such as response inhibition, contributes to the presence of both positive^15,19^ and negative^17,20^ symptoms. However, several studies have found no relationship between abnormal activation during response inhibition and symptoms^21,22^, and thus the degree of specificity of the neural substrate of symptoms to cognitive control related processing remains unclear.

A further open question concerns the degree to which the neural underpinnings of symptoms in schizophrenia change over the course of the illness. It is possible that the mechanisms underlying the initial development of symptoms during the early phases of the illness differ from those in the later more chronic maintenance phase^23^, and thus regions associated with psychotic symptoms are likely to vary through the course of the illness. Such a process may mirror habituation in the processing of perceptual stimuli or motor and cognitive learning, whereby a shift can be observed in the neural regions associated with early developmental and later more settled processing during the same task^24,25^. Moreover, these neural mechanisms may become harder to identify as the illness progresses as they are increasingly subject to the confounding effects of illness chronicity, medication exposure, and socio-environmental deprivation; as well as any neuroplasticity related to persistence of these symptoms^26,27^. For example, a correlation between disorganization symptoms and dorsolateral PFC activation has typically been observed during cognitive control tasks in drug-naïve schizophrenia patients^28^, but was not observed in medicated patients^17,21^. It has therefore been suggested that antipsychotic medication may alter the association between brain activity and symptoms^29^. This has implications for studies attempting to pinpoint how symptoms develop; many fMRI studies include chronic patients with schizophrenia who have been medicated for a number of years.

A classic approach to identifying symptom-related brain activation is to clarify the regions showing abnormal activation in patients compared to healthy controls, and then to test for correlations with symptoms specifically within these regions^29^. This method has the advantage of establishing a direct link between dysfunctional cognitive processing and clinical psychopathology. However, it relies on the presence of group differences in average activation, leading to a possible failure to identify associations in regions in which activation in patients does not differ from that in controls. Indeed, by its very nature, symptom heterogeneity can obscure group differences in average activation, such that etiologically relevant correlations between these symptom dimensions and neural activation patterns are not detected. This issue can be resolved by assessing associations between symptoms and neural activation on a whole-brain level independently of healthy control comparisons. The earliest studies took this approach with the use of resting state positron emission tomography (PET) by investigating associations between the severity of various symptom domains and regional cerebral blood flow (rCBF) in schizophrenia patients^30–33^ or by assessing the specificity of rCBF abnormalities to distinct symptom subgroups^34–36^. Throughout the last two decades, the focus has shifted increasingly toward the use of task-related fMRI, although only few studies report the relationship between brain activation and symptom severity irrespective of activation differences relative to healthy control groups^37–40^.

In the current study, we assess fMRI activation on a verbal Stroop task in a large sample of patients with early psychosis (EP) and a further sample of chronic patients with a diagnosis of schizophrenia (CHR-SZ). Previous studies have stressed the involvement of cognitive control in the development of both positive and negative symptoms; the role in maintenance of symptoms is less clear. Thus, we hypothesise an association between symptoms and activation in cognitive control related regions such as ACC and SMA/pre-SMA in EP patients rather than chronic patients. In an exploratory approach, we compare correlations between activation and symptoms between EP and CHR-SZ groups to address the question of changes in the neural substrate of symptoms throughout the course of illness. If cognitive control related mechanisms are primarily involved in the development, but not maintenance, of symptoms, correlations will no longer be observed in the chronic patient group. We analyse both activation that is specific to response inhibition (incongruent vs. congruent condition) as well as activation averaged across both task conditions, reflecting the more general effects of cognitive control and attentional processing.

## Method

### Participants

Ninety-five medicated early psychosis (EP) patients participated in the study. Inclusion criteria required patients to have experienced a first episode of psychosis within the previous five years, however the mean duration of illness was 1.7 years. In addition, 38 medicated chronic schizophrenia patients (CHR-SZ) were included (mean duration of illness 15.7 years). The CHR-SZ group consisted of treatment resistant and treatment responsive patients (matched for key demographic and clinical variables and thus relatively homogeneous aside from symptom severity) who were previously shown not to differ on the Stroop task^41^ and were thus collapsed into one group. Exclusion criteria for all patients were a history of neurological illness, current major physical illness, and drug dependency over the last six months. Positive and negative symptom severity was assessed by a trained researcher using the Positive and Negative Syndrome Scale (PANSS)^42^. Sample characteristics are presented in Table 1. All subjects provided written informed consent to take part the study and were compensated for their time and travel. The London Camberwell St Giles Research provided ethical approval and Ethics Committee and experiments were compliant with the Declaration of Helsinki.

**Table 1:** Sample characteristics of the early psychosis (EP) and chronic schizophrenia (CHR-SZ) patient groups.

	EP		CHR-SZ		*T*	*P*
	*M*	*SD*	*M*	*SD*		
Age	26.5	6.1	42.7	10.0	9.27	<0.001
WASI IQ	98.8	16.2	94.7	16.1	1.29	0.201
Onset age (years)	24.9	6.2	26.8	6.6	1.55	0.122
Illness duration (years)	1.7	1.3	15.6	9.4	9.02	<0.001
CPZ equivalents	244.0	144.1	367.0	222.7	3.13	0.002
**PANSS score**						
Positive symptoms	13.5	5.6	15.7	5.7	1.93	0.060
Negative symptoms	13.3	5.5	16.2	6.2	2.56	0.012
General symptoms	30.0	8.0	29.4	9.7	0.41	0.684
Total score	56.9	16.3	61.7	18.9	1.45	0.149
					*Χ* ^2^	*P*
Female (%)	15		42		3.01	0.083

### Procedure

Subjects performed a verbal Stroop paradigm while undergoing functional magnetic resonance imaging. A description of the task as well as imaging procedures and processing can be found in the Supplementary Materials.

### Behavioural data analysis

While behavioural performance was monitored during completion of the task to ensure that participants had understood the task instructions and were not responding randomly, reliable reaction times (RTs) from a number of trials could not be extracted from resulting audio files due to technical issues for some participants. Participants for whom RT data from more than 20 trials were unavailable were excluded from the behavioural analysis, resulting in a behavioural sample of 75 EP and 36 CHR-SZ patients. The reaction time (RT) Stroop effect (defined as mean RT in milliseconds on incongruent minus mean RT in milliseconds on congruent trials) as well as the Accuracy Stroop effect (defined as accuracy on congruent minus accuracy on incongruent trials) was assessed for each subject and compared between groups using t-tests.

### Imaging data analysis

Three EP subjects were excluded due to technical problems with the neuroimaging data, and a further four EP patients were excluded due to the absence of PANSS data, resulting in a final MRI sample of 88 EP and 38 CHR-SZ patients. The functional MRI data were analysed using the general linear model as implemented in FSL FEAT. For the first level analysis, regressors representing onsets of stimulus presentation for incongruent trials and congruent trials were included into the model. Each regressor was modelled as a delta function and convolved with a canonical hemodynamic response function. Standard and extended motion parameters, as well as a motion artefact confound matrix, which identified motion-corrupted volumes, were added as regressors of no interest. Volumes detected as corrupted were calculated by DVARS^43^ as implemented by FSL Motion Outliers. Contrasts of interest for the group level analysis were the *Stroop* contrast (Incongruent > Congruent) and the *Task* contrast (Incongruent + Congruent). Whole-brain activation was determined by a voxelwise z-threshold of 3.1 (*p* < 0.001 uncorrected) and a cluster significance threshold of p = 0.05 (whole-brain FWE corrected for multiple comparisons).

We examined significant activation across both groups as well as group differences in activation for both of these contrasts. Correlations between brain activation and symptoms were initially assessed within each group separately. This was done in order to increase power to detect group-specific associations between symptoms and brain activation. Next, group × symptom interactions were assessed on a whole-brain level as well as within regions of interest (ROIs) defined by the significant group-specific correlation clusters. The ROI approach was taken in order to avoid false interpretations about group differences in correlations (since the presence of an association in one group in the absence of that association in the other group does not automatically amount to a significant difference in the association between groups). Due to a significant group difference in chlorpromazine (CPZ) equivalent dosage and a marginally significant difference in sex distributions between the two groups, we tested whether regional effects from the group comparison analyses remained significant after controlling for CPZ equivalent dose and sex to exclude the possibility that findings were due to these possible confounds.

## Results

### Symptoms

The severity of positive and negative symptoms was somewhat higher in the CHR-SZ group compared to the EP group (Table 1), however this only reached significance in negative symptoms. There was no difference in total PANSS scores between the two groups. Importantly, both patient groups showed a similar range of PANSS positive (EP: min = 7, max = 35; CHR-SZ: min = 7, max = 26) and negative (EP: min = 7, max = 30; CHR-SZ: min = 7, max = 29) scores, indicating that correlative analyses within each group would cover a similar range of symptom severity. Mean symptom severity as well as frequency distributions of the individual positive and negative symptom subscales for each group are depicted in Supplementary Figs 2–5, indicting overall comparable symptom profiles in the two groups.

### Behavioural results

Behavioural data analyses were conducted on 75 EP and 36 CHR-SZ patients. A significant RT Stroop effect was observed both in EP (*M* = 141.70, *SD* = 105.05, *p* = < 0.001) and in CHR-SZ (*M* = 179.78, *SD* = 133.88, *p* = < 0.001) patients, but this did not differ between groups, *p* = 0.14. There was also a significant Accuracy Stroop effect in EP (*M* = 0.05, *SD = *0.07, *p* = < 0.001) and CHR-SZ (*M* = 0.07, *SD* = 0.08, *p* = < 0.001) patients, which did not differ significantly between groups, *p* = 0.17.

### Neuroimaging results

#### Mean activation

MRI analyses were conducted on 88 EP and 38 CHR-SZ patients.

On the *Stroop* (incongruent > congruent) contrast, significant activation was observed across both groups in regions classically associated with the Stroop task, including a midline section extending from ACC to supplementary motor area (SMA), bilateral precentral cortices, bilateral striatum and thalamus, and intracalcarine cortex. Significant deactivation was observed in right lateral occipital cortex (see Supplementary Fig. 1A). Exact regions can be found in Supplementary Table 1.

On the *Task* (incongruent + congruent) contrast, there was more widespread activation in areas including ACC/SMA and bilateral prefrontal cortical regions extending to precentral gyri, bilateral thalamus, putamen, brainstem, superior parietal cortices and cerebellum. Significant deactivations were observed in medial prefrontal cortex, bilateral superior frontal cortex, temporal poles, precuneus and lateral occipital cortices (see Supplementary Fig. 1B). These regions of deactivation are mostly consistent with areas associated with the default mode network, which is known to deactivate during task engagement. All significant regions of activation and deactivation are listed in Supplementary Table 2.

#### Group differences in activation

**Figure 1:**
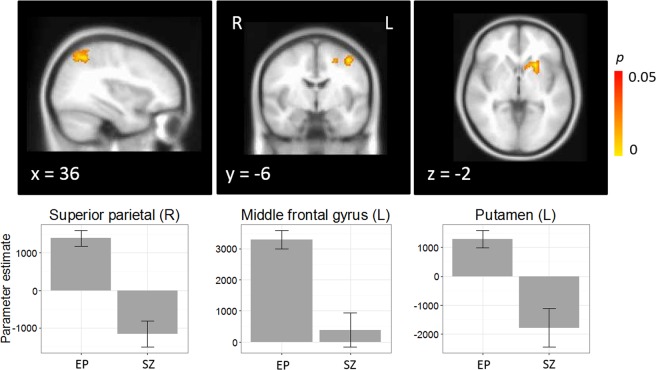
Main effect of group for the Task (incongruent + congruent) contrast, with peak activation in right superior parietal cortex [16, −54, 56], left middle frontal gyrus [−40, −6, 52], and left putamen [−22, 16, −2].

One-way analysis of variance did not reveal any differences in activation between EP and CHR-SZ patients for the *Stroop* contrast. However, there was a significant effect of group on the *Task* contrast in several regions. Specifically, across both trial conditions, EP showed greater activation than CHR-SZ in right superior parietal cortex (MNI: 16, −54, 56; 1197 voxels; *p* < 0.001), left middle frontal gyrus (MNI: −40, −6, 52; 330 voxels, *p* = 0.004), and left putamen (MNI: −22, 16, −2; 292 voxels; *p* = 0.007) (Fig. 1). Interestingly, this was due to significant activation in EP in all three regions, but deactivation in CHR-SZ in superior parietal cortex and putamen, and no significant activation in middle frontal gyrus. Group differences in these regions remained significant after controlling for CPZ equivalents and sex, all *p*s < 0.001.

#### Correlations with symptoms

There were no significant correlations between *Stroop* activation and either positive or negative symptoms in the EP or CHR-SZ patient group, nor was there a significant group × (positive or negative) symptom interaction at whole-brain level.

**Figure 2:**
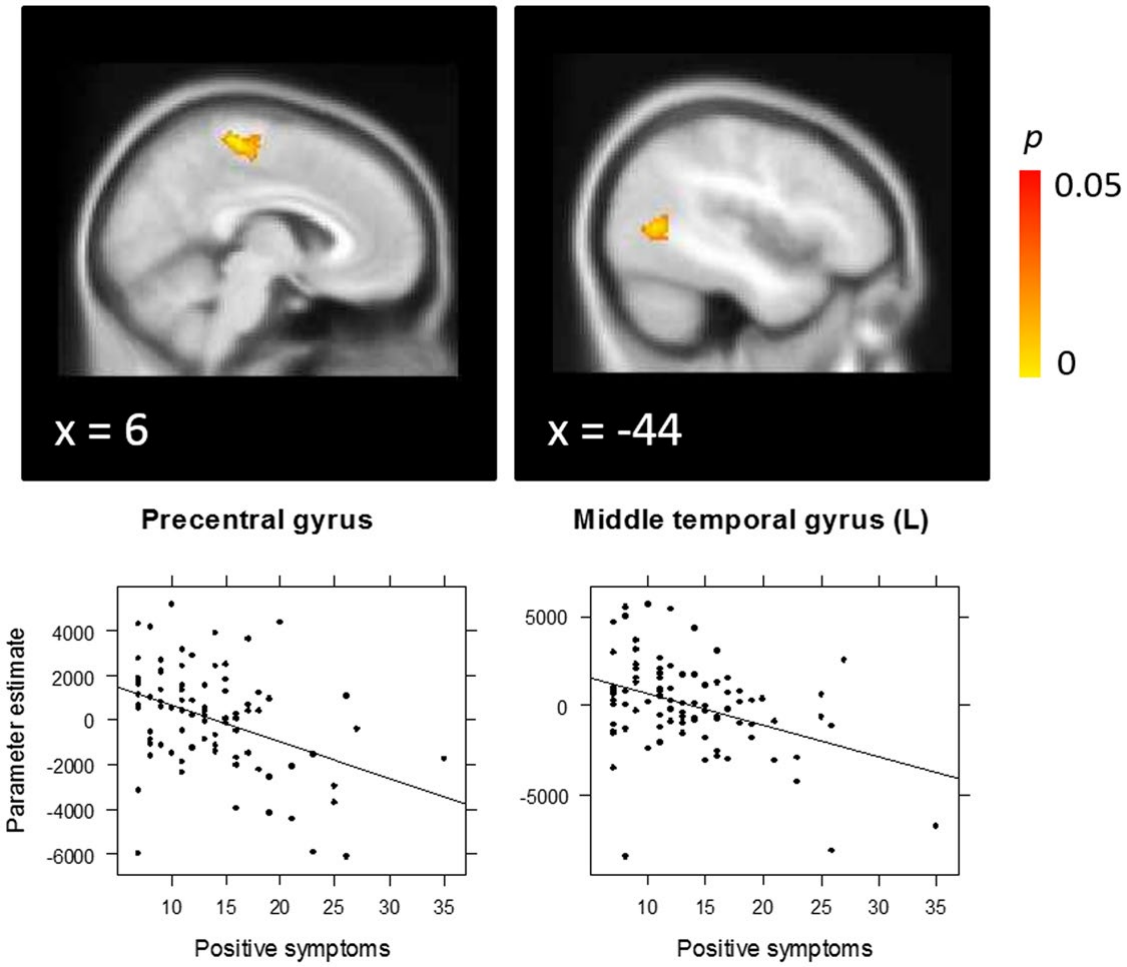
Correlation between positive symptoms and Task (incongruent + congruent) activation in early psychosis patients, with peak activation in precentral gyrus including dorsal supplementary motor area [2, −28, 62] and left middle temporal gyrus [−46, −62, 8].

On the *Task* contrast, EP patients showed a significant negative correlation between activation and positive symptoms in a midline precentral gyrus region including the dorsal SMA as well as left middle temporal gyrus (Fig. 2). Higher symptom severity was associated with reduced neural activation across both incongruent and congruent conditions in these regions.

**Figure 3:**
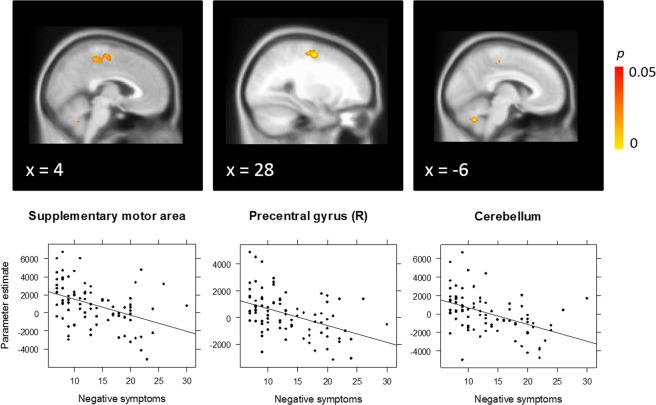
Correlation between negative symptoms and Task (incongruent + congruent) activation in early psychosis patients, with peak activation in the supplementary motor area [12, −12, 58], right precentral gyrus [30, −8, 56], and cerebellum [−6, −62, −40].

EP patients also showed a significant negative correlation between activation on the *Task* contrast and negative symptoms in dorsal SMA, right precentral gyrus, and cerebellum (Fig. 3). Again, higher symptom severity was associated with reduced activation in these regions across both task conditions.

No significant correlations between neural activation and (positive or negative) symptom severity were observed for the *Task* contrast in CHR-SZ patients.

**Figure 4:**
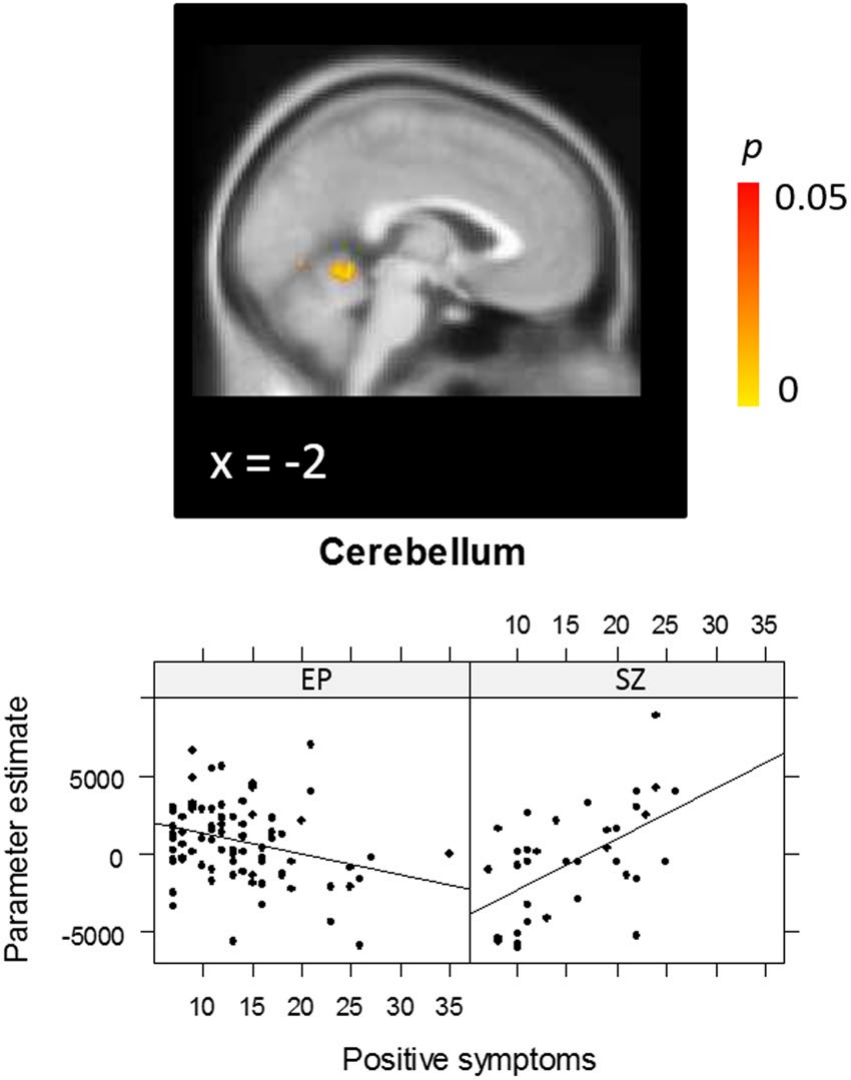
Group × positive symptom interaction for the Task (incongruent + congruent) contrast, with peak activation in the cerebellum [10, −46, −10].

A whole-brain analysis of the Group × positive symptom interaction on the *Task* contrast revealed a significant effect in a cluster extending from the cerebellum to lingual gyrus (MNI: 10, −46, −10; 770 voxels; *p* < 0.001) (Fig. 4). In this region, EP patients showed a negative correlation between positive symptoms and activation, whereas CHR-SZ patients showed a positive correlation. The interaction in this region remained significant after controlling for CPZ equivalent medication dose and sex, *p* < 0.001.

Next we tested for Group × symptom interactions in the significant correlation clusters observed in the EP group, in order to ascertain whether correlations in these regions specifically were significantly different in the CHR-SZ group. For positive symptoms, there was a significant interaction both in the midline precentral region including the dorsal SMA (beta = 176.26, *p* = 0.026), as well as the middle temporal gyrus region (beta = 231.68, *p* = 0.009), indicating that the correlation between positive symptoms and neural activation differed significantly between groups. While EP showed a negative correlation between symptoms and activation in these regions (precentral gyrus/SMA: *R* = −0.40, *p* < 0.001; middle temporal gyrus: *R* = −0.39, *p* < 0.001), a non-significant positive correlation was observed in CHR-SZ patients (precentral: *R* = 0.03, *p* = 0.872; middle temporal gyrus: *R* = 0.12, *p* = 0.493). The interaction terms in these regions remained significant after controlling for CPZ equivalent medication dose and sex, all *p*s < 0.05. For negative symptoms, a significant interaction was only observed in the cerebellar cluster (beta = 226.14, *p* = 0.004). Again, while there was a negative correlation in the EP group (*R* = −0.47, *p* < 0.001), there was a non-significant correlation in the CHR-SZ group (*R* = 0.09, *p* = 0.600). The interaction in this region remained significant after controlling for CPZ equivalent medication dose and sex, *p* < 0.05. In contrast, no significant group × negative symptom interaction was observed in the SMA or precentral gyrus, indicating that the negative correlations between negative symptom severity and activation in these regions did not differ significantly between EP (SMA: *R* = −0.42, *p* < 0.001; precentral gyrus: *R* = −0.45, *p* < 0.001) and CHR-SZ patients (SMA: *R* = −0.14, *p* = 0.401; precentral gyrus: *R* = −0.09, *p* = 0.622).

## Discussion

In this study, we assessed whole-brain activation on a Stroop task and correlations with symptom severity in a large sample of early psychosis (EP) patients compared to a sample of chronic schizophrenia (CHR-SZ) patients. Subjects showed overall activation in regions consistent with a cognitive control network including ACC, SMA, bilateral motor cortices, thalamus and striatum. The key finding of this study is a differential correlation between positive symptom severity and activation in SMA, middle temporal gyrus and cerebellum in EP and CHR-SZ patients, as well as between negative symptom severity and activation in the cerebellum in the two groups. Activation was inversely related to symptom severity in EP patients, but was not related to symptoms in chronic patients, with the exception of cerebellar activation, which showed a positive correlation with positive symptoms in CHR-SZ. These results suggest that the extent to which cognitive control related mechanisms are associated with symptoms differs as a function of illness chronicity, as is discussed in more detail below.

In the between-group comparison of activation, chronic patients showed reduced overall task activation across both trial types compared to EP patients in left middle frontal gyrus, right superior parietal cortex and left putamen, regions known to be important in cognitive control^44^. The findings suggest that activity in this functional network may decrease over the illness course, consistent with previous findings that Stroop-related activation is negatively related to number of psychotic episodes experienced^45^.

We observed a significant association between positive symptom severity and reduced activation in medial precentral gyrus including the dorsal SMA and left posterior middle temporal gyrus in EP. The left middle temporal gyrus is relevant for semantic control during language generation^46,47^ and is also known to be involved in resolution of semantic interference during overt responding^48^. Recent research suggests that similar mechanisms underlie semantic interference effects and classic Stroop colour-word interference^49^. Additionally, left middle temporal gyrus has been repeatedly implicated in schizophrenia, with reduced volume observed compared to healthy controls in chronic schizophrenia patients as well as first episode psychosis patients and their unaffected siblings^50–52^. Middle temporal gyrus activation has been observed during auditory hallucinations^53,54^ and functional as well as structural abnormalities have been associated with the severity of hallucinatory symptoms^50,55^. Activation of medial sensorimotor and premotor cortices is commonly observed in the Stroop task and is likely involved in error monitoring when exerting cognitive control^56^. Interestingly, SMA dysfunction has also been associated with alien limb syndrome, whereby movements are experienced as occurring independently of an individual’s own active control^57,58^. This is suggested to result from an inability to suppress implicitly activated motor programmes, normally controlled by SMA regions^59^. The resulting failure to distinguish internally and externally generated movement is akin to delusions of control commonly observed in psychosis. Our findings suggest that abnormal functioning of the SMA and middle temporal regions in the context of cognitive control may provide an important marker of positive symptom severity during the development stage rather than chronic stage of psychotic illness. We did not observe a correlation between symptoms and the region most classically associated with cognitive control, the anterior cingulate cortex (ACC). This relates to a meta-analytic review of neural activation during executive function tasks in schizophrenia, which showed abnormal ACC activation in patients across all tasks, but more dorsal effects closer to the SMA for the Stroop task specifically^1^.

With respect to negative symptoms, we observed an inverse relationship with activation in SMA, right precentral gyrus and cerebellum in EP. Similar to the correlations with positive symptoms, this association indicates that EP patients with severe negative symptoms are less able to recruit these regions while exerting cognitive control. Negative symptoms have long been associated with cognitive dysfunction in schizophrenia^60^ and several authors have emphasised the role of diminished cognitive control in the failure to learn reward outcomes, ultimately leading to motivational subcomponents of negative symptomatology such as anhedonia and avolition^61,62^. Interestingly, the correlation in SMA and precentral gyrus did not differ significantly from that in chronic patients, suggesting that the cortical neural substrate of negative symptoms remains more stable over the illness course, in contrast to that of positive symptoms.

A striking finding in this study is that of reduced cerebellar activation with increasing (positive and negative) symptom severity in EP patients, but increased cerebellar activation with more severe symptoms in the CHR-SZ group. The traditional, limited view of the cerebellum as being primarily involved in motor or associative learning has been revised in recent decades with the accumulation of evidence showing that the cerebellum is involved in a multitude of higher cognitive processes^63,64^. There is an increasing consensus that the cerebellum acts as a coordinator or modulator of cortical activity via a cortico-cerebellar-thalamo-cortical circuit (CCTCC). CCTCC dysfunction in schizophrenia, driven or contributed to by cerebellar abnormalities, has been suggested to result in poorly coordinated mental activity (“cognitive dysmetria”), which may underlie a range of symptoms and cognitive abnormalities^65,66^. Underscoring a potential causal role of the cerebellum in symptom development, several recent interventional studies have shown that application of repetitive transcranial magnetic stimulation to the cerebellar vermis results in a significant improvement of negative symptom severity in patients with schizophrenia^67–69^.

Our findings suggest that the role of the cerebellum with respect to symptoms differs in the early and late stages of psychosis. The observation is partly mirrored in findings from previous PET research showing a positive correlation between cerebellar rCBF and both positive and negative symptoms in chronic schizophrenia patients^70^, but a negative correlation with negative symptoms observed in neuroleptic-naive first episode psychosis (FEP) patients by the same group^71^. Increased cerebellar activation in conjunction with increased symptom severity was interpreted by the authors as compensatory activity in response to decreases in other regions of the CCTCC. This interpretation is in line with reduced cortical and subcortical activation observed in our study in chronic patients compared to EP patients with relatively preserved task performance. Both hyper- and hypoactivations of the cerebellum have been observed in schizophrenia on a range of tasks including working memory and affective processing^72–74^, providing further support for a dynamically changing role of the cerebellum in psychosis.

It is important to highlight that all neuroimaging effects observed in this study relate to the *Task* contrast, which averages over both incongruent and congruent trials. Thus, the observed activation changes associated with this contrast are not specific to response inhibition; however they likely nevertheless reflect effects of cognitive or attentional control more generally. As can be seen in Supplementary Fig. 1, activation associated with the *Task* contrast is highly similar to that associated with the *Stroop* contrast, with some additional activation in parietal, occipital and cerebellar regions. While it is reasonable to assume that the *Stroop* contrast taps into response conflict specifically, it is clear that congruent trials are not free from cognitive control (particularly in task setups with randomly interspersed trial types) and as such subtracting these out may obscure effects relevant for control related processes^75^.

A limitation of this study is the unequal sample sizes of the EP and CHR-SZ groups. It is possible that the EP sample was more sensitive to correlation effects due to a larger number of participants. However, the CHR-SZ consisted of a reasonably large sample of 38 patients, providing over 80% power to detect a comparable correlation of 0.4 as observed in the EP sample. We are therefore confident that our analyses did not miss a clinically meaningful true correlation between brain activation and symptom severity. In this study we cannot rule out a potential treatment effect caused by long-term medication exposure in the chronic patient group, who were also receiving a higher dosage of antipsychotic medication at the time of study. This is a common limitation inherent to research in chronic medicated patients, further emphasising the importance of studying neural correlates of symptoms early on in the illness. However, controlling for current medication dosage did not significantly alter the observed group differences, suggesting that this unlikely confounded our findings. A related issue pertains to the heterogeneity of the studied samples. The chronic cohort consisted of patients with a diagnosis of schizophrenia who had responded to antipsychotic treatment to variable extents. The early psychosis cohort, in contrast, were early on in their illness trajectory, and it is possible that a number of these patients will not go on to receive a diagnosis of schizophrenia. Future research will benefit from within-subject longitudinal follow-up designs in order to address the role of illness chronicity in the brain-symptom relationship more reliably. Finally, given the correlative nature of fMRI, it is not possible to infer a definitive causal relationship between cognitive control related neural mechanisms and symptom development. However, understanding the spatial distribution of the neural correlates of symptoms can provide insights into potential associated mechanisms and thereby offer a basis for future causal investigations such as non-invasive intervention studies. Future research could also usefully examine changes in functional connectivity beyond simple activation in order to establish whether a breakdown of network connectivity is involved in symptom development and maintenance.

Overall the results of this study suggest that positive symptoms are associated with abnormalities in cognitive control related circuits in the early stages of psychosis, but once stabilised are no longer tightly coupled with mechanisms underlying cognitive control. In contrast, the cortical neural substrate of negative symptoms appears to remain more stable over the psychotic illness course. Finally, the findings suggest that initial formation and later maintenance of positive and negative symptoms may be associated with opposite mechanisms in the cerebellum. These results bear implications not only for the mechanistic understanding of symptoms but also research into potential targets for novel treatments, since different mechanisms may need to be targeted depending on the stage of psychotic illness.

## Supplementary information

Supplementary material

## Data Availability

Data and code are available from the corresponding author upon reasonable request.
