# Supplementary material for: Neural correlates of positive and negative symptoms through the illness course: an fMRI study in early psychosis and chronic schizophrenia
Source: Sci Rep. 2019 Oct 8;9:14444. doi: 10.1038/s41598-019-51023-0 (PMC6783468; doi:10.1038/s41598-019-51023-0)
Supplement: Supplementary file 1 — Supplementary material [file 41598_2019_51023_MOESM1_ESM.pdf]

__Supplementary material__

Neural correlates of positive and negative symptoms through the illness course: an fMRI study in early psychosis and chronic schizophrenia

Vanes, L.D., Mouchlianitis, E., Patel, K., Barry, E., Wong, K., Thomas, M., Szentgyorgyi, T., Joyce, D., & Shergill, S.

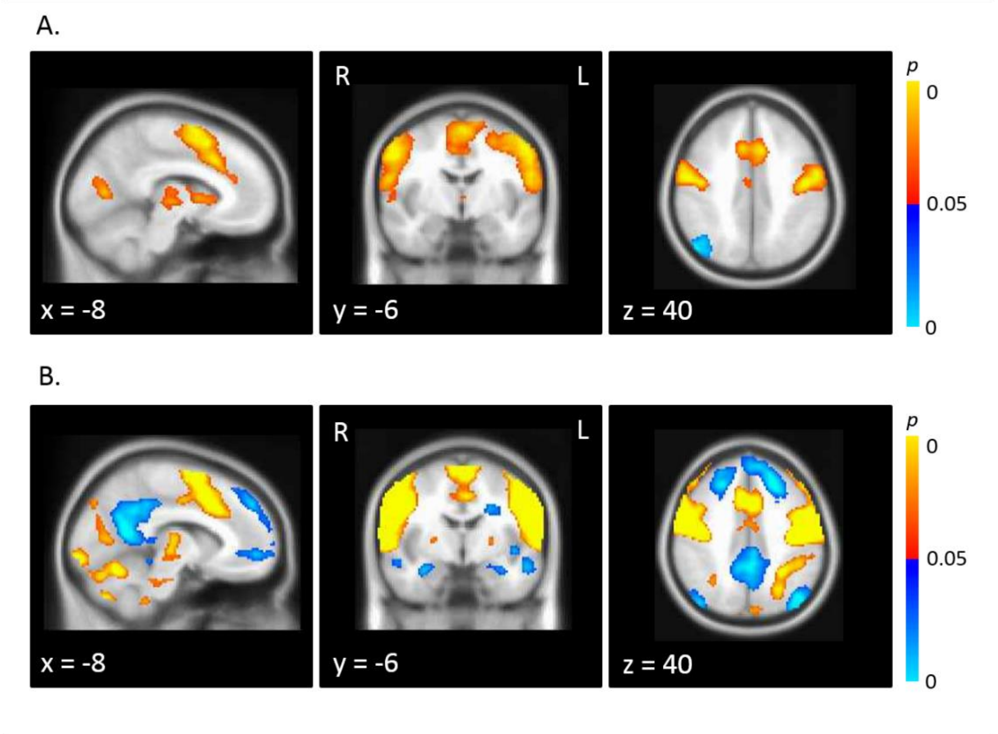

Supplementary Figure 1. *Mean activation (red) and deactivation (blue) across both groups for A. the Stroop contrast (incongruent > congruent) and B. the Task contrast (incongruent + congruent)*

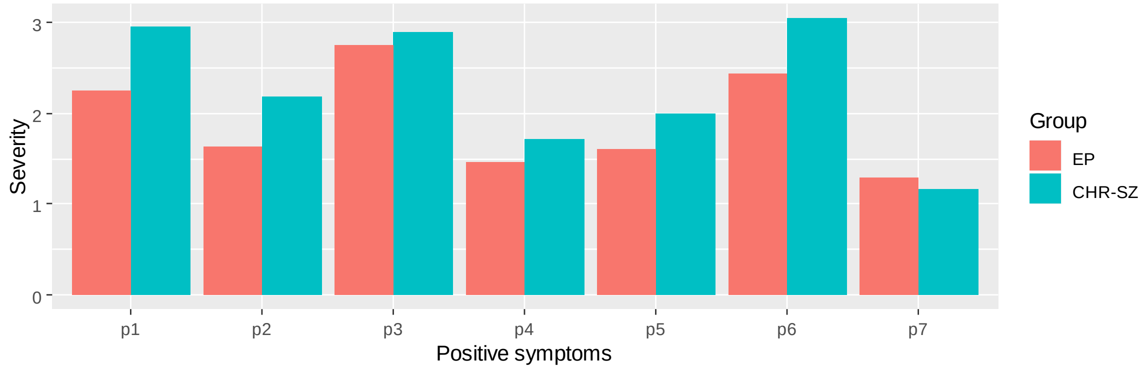

Supplementary Figure 2. *Mean symptom severity by positive symptom subscale in early psychosis (EP) and chronic schizophrenia (CHR-SZ) patients.*

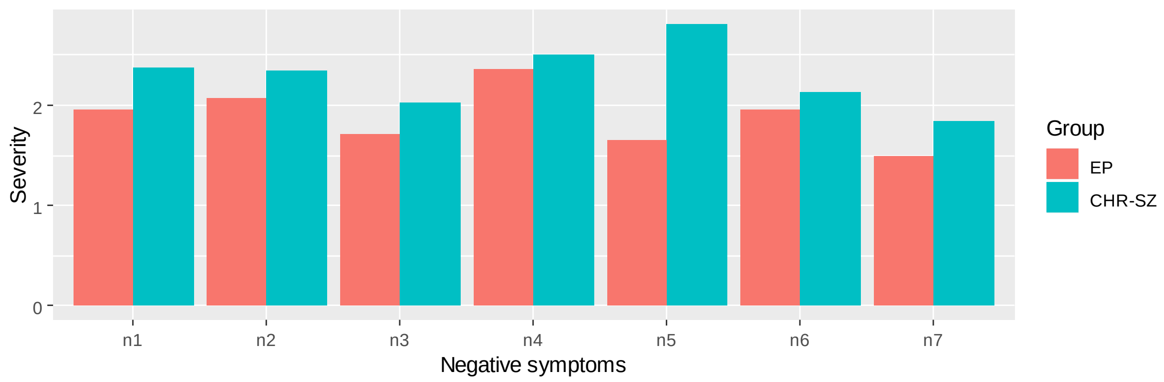

Supplementary Figure 3. *Mean symptom severity by negative symptom subscale in early psychosis (EP) and chronic schizophrenia (CHR-SZ) patients.*

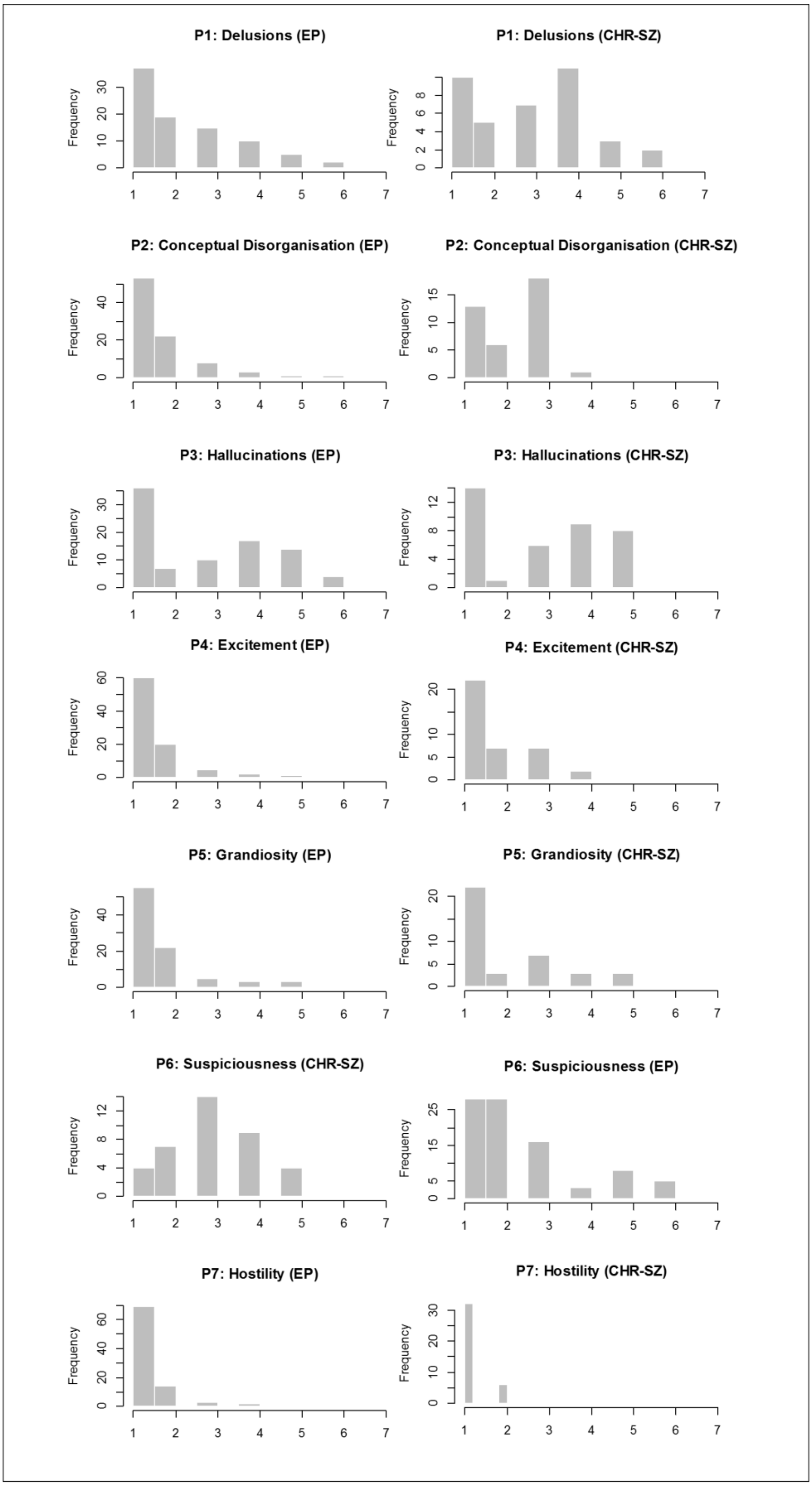

Supplementary Figure 4. *Distribution of positive symptom subscale severities in early psychosis (EP) and chronic schizophrenia (CHR-SZ) patients.*

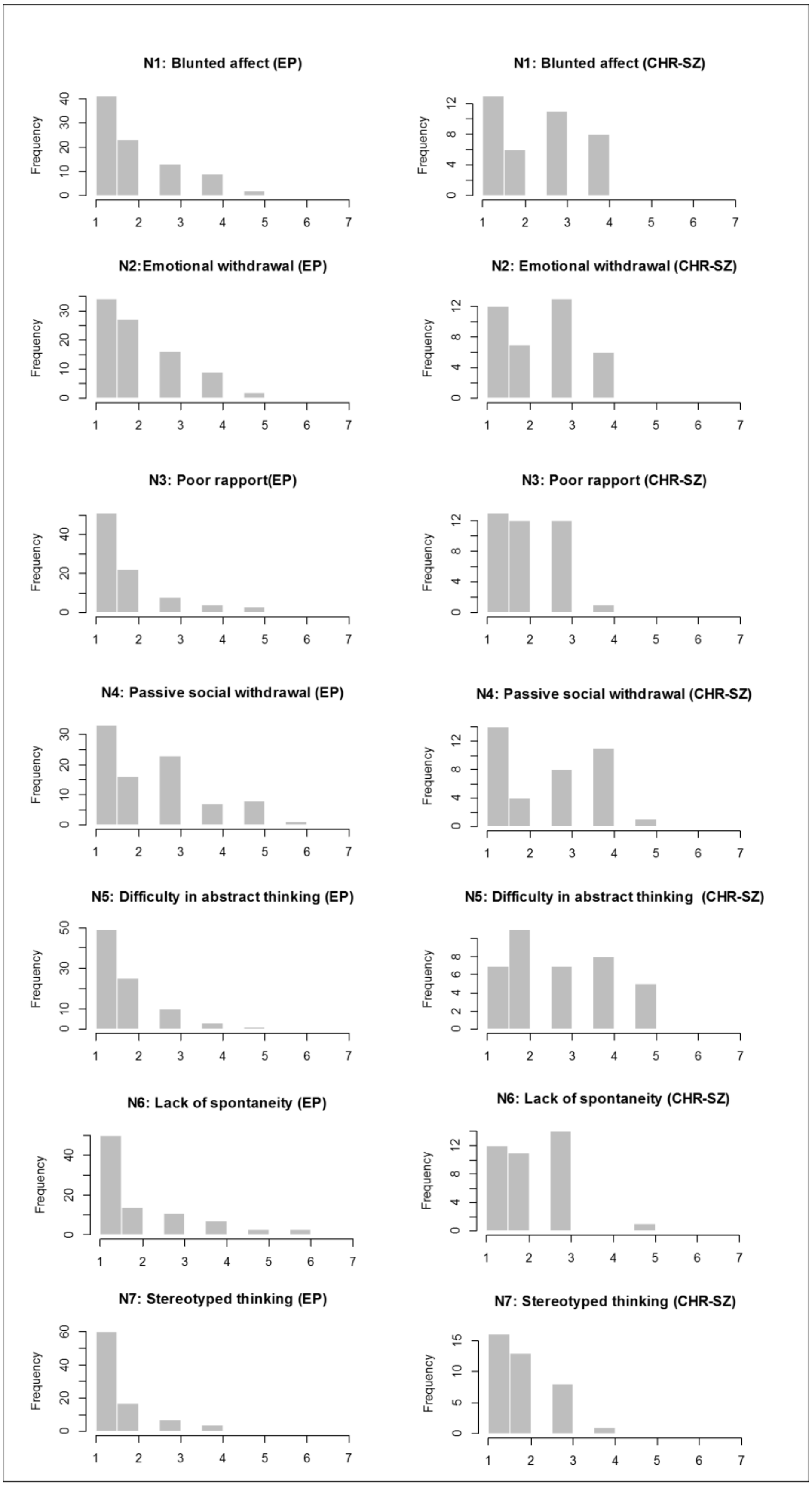

Supplementary Figure 5. *Distribution of negative symptom subscale severities in early psychosis (EP) and chronic schizophrenia (CHR-SZ) patients.*

Supplementary Table 1. *Significant clusters of activation and deactivation associated with the Stroop (incongruent vs. congruent) contrast*

Region	Side	k	z	MNI		
**Activation**						
Supplementary motor area	L	9162	6.89	-2	2	62
Anterior cingulate cortex	R			6	2	62
Superior frontal gyrus	L			-6	12	54
Precentral gyrus	L			-46	-14	40
Precentral gyrus	L			-54	-4	44
Precentral gyrus	L			-52	-10	48
Precentral gyrus	R	3554	6.36	56	-6	42
Precentral gyrus	R			58	-4	46
Precentral gyrus	R			46	-12	38
Precentral gyrus	R			62	-2	20
Frontal operculum cortex	R			36	16	10
Frontal operculum cortex	R			46	16	-2
Thalamus	L	579	4.81	-14	-18	0
Thalamus	R			16	-18	0
Thalamus	R			16	-18	2
Thalamus	L			-8	-16	10
Thalamus	R			4	-20	2
Thalamus	R			12	-16	10
Precentral gyrus	L	396	5.33	-20	-28	64
Precentral gyrus	R	277	4.83	22	-28	60
Intracalcarine cortex	L	393	4.98	-8	-74	10
Lingual gyrus	L			-2	-78	10
Cuneal cortex	L			-6	-82	20
Intracalcarine cortex	L			-22	-62	6
Cuneal cortex	R			2	-86	22
**Deactivation**						

Lateral occipital cortex	R	776	4.86	40	-74	44
Lateral occipital cortex	R			52	-62	28
Lateral occipital cortex	R			52	-72	30
Lateral occipital cortex	R			48	-76	32
Angular gyrus	R			44	-56	24
Lateral occipital cortex	R			42	-60	48

Supplementary Table 2. *Significant clusters of activation and deactivation associated with the Task (incongruent + congruent) contrast*

Region	Side	k	z	MNI		
**Activation**						
Frontal pole	L	562	4.72	-2	60	-8
Medial frontal cortex	L			-2	52	-10
Anterior cingulate cortex	L			-2	44	-6
Frontal pole	L			-2	66	2
Putamen	R	351	5.1	20	6	6
Precentral gyrus	R	38738	8.21	64	-2	16
Precentral gyrus	L			-48	-10	30
Postcentral gyrus	L			-42	-14	34
Occipital pole	L			-32	-96	-12
Lateral occipital cortex	L			-38	-90	-12
Putamen	L	232	4.35	-22	-2	4
Caudate	L			-14	6	8
Putamen	L			-18	6	8
Caudate	L			-16	0	14
White matter	L	260	4.88	-22	-10	32
White matter	L			-28	-20	30
Thalamus	R	261	5.56	12	-16	6
Thalamus	R			12	-18	-2
Thalamus	L	378	5.75	-12	-18	6
Thalamus	L			-12	-30	-2
Thalamus	L			-6	-16	-10
Precentral gyrus	L	443	6.11	-18	-30	62
Precentral gyrus	L			-22	-26	74
Postcentral gyrus	L			-20	-38	76
Postcentral gyrus	L			-36	-30	70
Postcentral gyrus	L			-30	-38	72

Postcentral gyrus	R	389	6.18	20	-30	64
Postcentral gyrus	R			24	-30	74
Precentral gyrus	R			22	-24	72
Brain stem	R	639	5.91	2	-34	-46
Brain stem	R			6	-30	-30
Brain stem	R			2	-34	-30
Brain stem	R			-2	-24	-30
Brain stem	R			-8	-20	-28
Brain stem	R			-10	-32	-34
Supramarginal gyrus	R	609	4.96	58	-40	54
Lateral occipital cortex	R			32	-60	46
Superior parietal cortex	R			34	-56	50
Supramarginal gyrus	R			50	-40	48
Lateral occipital cortex	R			36	-66	60
Angular gyrus	R			48	-52	60
Lateral occipital cortex	L	1982	6.64	-30	-62	50
Superior parietal cortex	L			-36	-46	46
Superior parietal cortex	L			-28	-54	46
Supramarginal gyrus	L			-48	-46	52
Supramarginal gyrus	L			-54	-38	50
Lateral occipital cortex	L			-26	-72	36
Supracalcarine cortex	R	960	5.18	2	-78	18
Intracalcarine cortex	R			12	-70	12
Intracalcarine cortex	L			-10	-74	10
Intracalcarine cortex	L			-18	-70	8
Lateral occipital cortex	L			-8	-84	42
Cuneus	L			-2	-86	40
**Deactivation**						
Superior frontal gyrus	L	1790	6.04	-22	32	46
Superior frontal gyrus	L			-22	30	42

Frontal pole	L			-20	40	42
Superior frontal gyrus	L			-24	24	46
Superior frontal gyrus	L			-2	52	36
Frontal pole	L			-10	50	40
Superior frontal gyrus	R	814	5.72	22	30	48
Frontal pole	R			26	42	40
Temporal pole	R	330	5.06	42	18	-28
Middle temporal gyrus	R			52	0	-26
Temporal pole	R			50	8	-28
Superior temporal gyrus	R			54	-4	-14
Middle temporal gyrus	R			56	0	-20
Middle temporal gyrus	R			62	4	-20
Temporal pole	L	678	5.51	-48	4	-30
Middle temporal gyrus	L			-52	-4	-18
Temporal pole	L			-40	18	-30
Insula	L			-42	-4	-4
Temporal pole	L			-40	20	-24
Temporal pole	L			-40	14	-18
Precuneus	L	5992	6.38	-6	-56	14
Posterior cingulate cortex	L			-2	-46	38
Posterior cingulate cortex	L			-4	-40	40
Hippocampus	L			-26	-16	-16
Hippocampus	R			34	-10	-20
Lateral occipital cortex	R	802	5.59	54	-70	30
Lateral occipital cortex	R			42	-80	34
Lateral occipital cortex	R			48	-74	36
Lateral occipital cortex	R			48	-70	34
Lateral occipital cortex	L	1234	6.46	-42	-76	34
Lateral occipital cortex	L			-42	-76	38
Angular gyrus	L			-38	-54	26

Lateral occipital cortex	L			-60	-64	26
Precuneus	L	5992	6.38	-6	-56	14
Posterior cingulate cortex	L			-2	-46	38

Supplementary methods

Procedure

Subjects performed a verbal Stroop paradigm while undergoing functional magnetic resonance imaging. The screen was viewed via a head-mounted mirror. On each trial, a single colour word was presented on the screen ("BLUE", "RED", "GREEN", or "YELLOW") against a black background. The word was printed in one of four possible font colours (blue, red, green, or yellow). Word meaning and font colour were either congruent or incongruent, and subjects were instructed to respond verbally to the font colour and to ignore the word meaning. In addition, on fixation trials, a central white fixation cross was presented in the centre of the screen and no response was required. Thirty-three congruent, 33 incongruent, and 34 fixation trials were presented in randomised order, each with a duration of 700ms and inter-stimulus-interval of 2300ms. Responses were recorded via a microphone mounted inside the scanner in order to assess reaction times. Only responses between 200 and 2700 ms after stimulus presentation were recorded.

Scanning parameters

Functional scans were acquired using a T2* echo planar sequence (153 volumes, TR=2000 ms, TE=30 ms, field of view = 21.1 cm, slice thickness = 3 mm, matrix = 64 x 64, flip angle = 75°) sensitive to blood oxygenation level-dependent (BOLD) contrast on a 3T GE Excite II MR scanner (GE Healthcare, USA). A structural image was acquired for each subject with a T1-weighted magnetization prepared rapid acquisition gradient echo (MP RAGE) sequence (TR=7321 ms, TE=3 ms, TI = 400 ms, field of view = 240, slice thickness = 1.2 mm, 196 slices).

Imaging data preprocessing

The fMRI data was preprocessed and analysed using the FEAT tool from the FMRIB Software Library (FSL, http://fsl.fmrib.ox.ac.uk/fsl/fslwiki/, Smith et al. 2004). Functional and structural brain images were extracted from non-brain tissue using FSL's brain extraction tool (BET), and EPI images were realigned using MCFLIRT to correct effects of head motion. A 100-s temporal high-pass filter was applied and data was spatially smoothed using a Gaussian kernel of 5mm FWHM.
